# Dose Selection for an Adjuvanted Respiratory Syncytial Virus F Protein Vaccine for Older Adults Based on Humoral and Cellular Immune Responses

**DOI:** 10.1128/CVI.00157-17

**Published:** 2017-09-05

**Authors:** Judith Falloon, H. Keipp Talbot, Craig Curtis, John Ervin, Diane Krieger, Filip Dubovsky, Therese Takas, Jing Yu, Li Yu, Stacie L. Lambert, Tonya Villafana, Mark T. Esser

**Affiliations:** a MedImmune, Gaithersburg, Maryland, USA; b Vanderbilt University Medical Center, Nashville, Tennessee, USA; c Compass Research, Orlando, Florida, USA; d Center for Pharmaceutical Research, Kansas City, Missouri, USA; e Miami Research Associates, Miami, Florida, USA; f MedImmune, Mountain View, California, USA; Vanderbilt University Medical Center

**Keywords:** respiratory syncytial virus, vaccine, adult, adjuvant, cell-mediated immunity, TLR-4 agonist

## Abstract

This is the second phase 1 study of a respiratory syncytial virus (RSV) vaccine containing RSV fusion protein (sF) adjuvanted with glucopyranosyl lipid A (GLA) in a squalene-based 2% stable emulsion (GLA-SE). In this randomized, double-blind study, 261 subjects aged ≥60 years received inactivated influenza vaccine (IIV), a vaccine containing 120 μg sF with escalating doses of GLA (1, 2.5, or 5 μg) in SE, or a vaccine containing 80 μg sF with 2.5 μg GLA in SE. Subjects receiving 120 μg sF with 2.5 or 5 μg GLA were also randomized to receive IIV or placebo. Immunity to RSV was assessed by detection of microneutralizing, anti-F immunoglobulin G, and palivizumab-competitive antibodies and F-specific gamma interferon enzyme-linked immunosorbent spot assay T-cell responses. Higher adjuvant doses increased injection site discomfort, but at the highest dose, the reactogenicity was similar to that of IIV. Significant humoral and cellular immune responses were observed. The 120 μg sF plus 5.0 μg GLA formulation resulted in the highest responses in all subjects and in older subjects. These results confirm previous observations of vaccine tolerability, safety, and immunogenicity and were used to select the 120 μg sF plus 5.0 μg GLA formulation for phase 2 evaluation. (This study has been registered at ClinicalTrials.gov under registration no. NCT02289820.)

## INTRODUCTION

Respiratory syncytial virus (RSV) is well-known as a cause of disease in infants and immunosuppressed adults, but its role in disease in older adults has been less widely appreciated (1, 2). Disease caused by RSV in adults occurs during influenza season and is clinically indistinguishable from that caused by influenza virus (3). Testing for RSV is not widely implemented in adults, so cases often are not identified (3, 4). Recognition of the importance of RSV as an adult pathogen was led by Falsey et al. in the United States, and data now confirm its importance across the globe (2, 5). The burden of RSV disease in adults can be similar to that of influenza (6, 7); hence, a vaccine is greatly needed.

The severity of RSV disease by age manifests as the U-shaped curve that has been observed for other infectious diseases, with peaks in hospitalization rates being found for the very young and the very old (8, 9). The hospitalization rate begins to rise at about 50 years of age (9). Increasing rates of comorbidities, particularly cardiopulmonary disease, contribute to this pattern, but immunosenescence is a specific challenge in vaccine development in this age group. While their anti-RSV-neutralizing antibody titers are similar to those in younger adults, older adults are deficient in cellular immunity to RSV (10). Neutralizing antibodies are of known importance in the prevention of RSV in adults; however, cellular immunity is thought to be important in virus clearance (1, 11). Antibodies generated by RSV infection are short-lived and provide only limited protection, so reinfection with RSV is common throughout life (12–14). Both the short duration of humoral immunity and the limited cellular immune responses in adults suggest that a vaccine for this population may be more effective if it includes an adjuvant that boosts neutralizing antibodies and elicits RSV F-specific T cells.

We present the results of the second study of a vaccine containing soluble RSV fusion protein (sF) in the postfusion configuration and a Toll-like receptor 4 (TLR-4) agonist adjuvant, glucopyranosyl lipid A (GLA), in a squalene-based stable emulsion (SE), which was designed to promote a TH1-biased immune response (15–18). There has been general agreement that an RSV vaccine for older adults should contain the F protein, because it induces neutralizing antibodies and is conserved across the RSV A and B subtypes and over time, so that annual reformulation would not be necessary (19, 20). The inclusion of adjuvant was shown in a previous study to increase both cellular and humoral immunogenicity (21). The primary objective of this phase 1b study was to assess the safety and tolerability of RSV formulations containing escalating doses of GLA and to provide safety and immunogenicity data for use in dose selection for a phase 2 efficacy study (ClinicalTrials.gov registration no. NCT02508194).

(This work was presented in abstract form at the RSV16 10th International Respiratory Syncytial Virus Symposium, 28 September to 1 October, Patagonia, Argentina; RSV Vaccines for the World, 18 to 20 November 2015, La Jolla, CA; and The Macrae Foundation's XVIII International Symposium on Respiratory Viral Infections, 31 March to 2 April 2016, Lisbon, Portugal.)

## RESULTS

### Participants.

As planned, 264 subjects were randomized in January and February 2015; 261 were dosed, and 246 (94.3%) completed the study (see Fig. S1 in the supplemental material). The median age of the subjects was 68 years (range, 60 to 91 years), 42.5% were >69 years of age, and 8% were >80 years of age; 54.4% of the subjects were female, 92.7% were of the white race, and 18.4% were of Latino ethnicity (Table S1). One subject was immunosuppressed at enrollment and thus was excluded from the immunogenicity analysis population.

### Safety.

GLA increased local pain and tenderness in a dose-dependent fashion, but the solicited symptoms were low grade (only 1 severe symptom—headache—was reported, in an inactivated influenza vaccine [IIV] recipient) and of short duration (Table 1 and S2). At the highest level tested, 120 μg of sF with 5 μg of GLA, the solicited symptom profile was similar to that of influenza vaccine; for both vaccines, the median symptom duration was 2 days. In adverse event (AE) data through day 29, 2 grade 3 events—coronary artery disease and breast cancer—were reported. There were no serious AEs (Table S3), new-onset chronic diseases (Table S3), or AEs of special interest (AESI) considered by the investigators to be related to study dosing. The 1 AESI reported (considered unrelated to vaccine dosing) was asymptomatic uveitis in a subject who received a vaccine containing 120 μg of RSV sF with 5 μg of GLA and who was diagnosed with uveitis 15 days later when she had a scheduled evaluation that included optical coherence tomography prior to cataract surgery. Her vision was stable, she remained asymptomatic, and the event resolved.

**TABLE 1:** Local solicited symptoms reported during days 1 to 7^*d*^

Local solicited symptom	No. (%) of patients					
	Placebo^*a*^ (*n* = 108)	IIV^*b*^ (*n* = 122)	RSV vaccine			
			Cohort 4 (80 μg sF + 2.5 μg GLA; *n* = 20)	Cohort 1 (120 μg sF + 1 μg GLA; *n* = 39)	Cohort 2^*c*^ (120 μg sF + 2.5 μg GLA; *n* = 78)	Cohort 3^*c*^ (120 μg sF + 5 μg GLA; *n* = 78)
Any local solicited symptom	27 (25.0)	61 (50.0)	8 (40.0)	11 (28.2)	38 (48.7)	42 (53.8)
Tenderness or soreness at injection site	21 (19.4)	53 (43.4)	7 (35.0)	9 (23.1)	33 (42.3)	35 (44.9)
Pain at injection site	18 (16.7)	30 (24.6)	6 (30.0)	6 (15.4)	22 (28.2)	23 (29.5)
Swelling at injection site	1 (0.9)	3 (2.5)	0	1 (2.6)	1 (1.3)	4 (5.1)
Redness at injection site	1 (0.9)	3 (2.5)	0	1 (2.6)	3 (3.8)	0

a The placebo group includes subjects who received placebo in the IIV plus placebo groups in cohorts 2 and 3.

b The IIV group includes subjects who received IIV in cohort 1 and cohort 4 and IIV in the IIV plus placebo groups in cohorts 2 and 3.

c In this cohort, subjects received the RSV vaccine plus the IIV or RSV vaccine plus placebo.

d GLA, glucopyranosyl lipid A; IIV, inactivated influenza vaccine; RSV, respiratory syncytial virus; SE, stable emulsion; sF, soluble RSV fusion protein. One subject reported postdose swelling but did not report a measurement on days 4 and 5, so her highest severity grade could not be determined.

### Immunogenicity.

**FIG 1:**
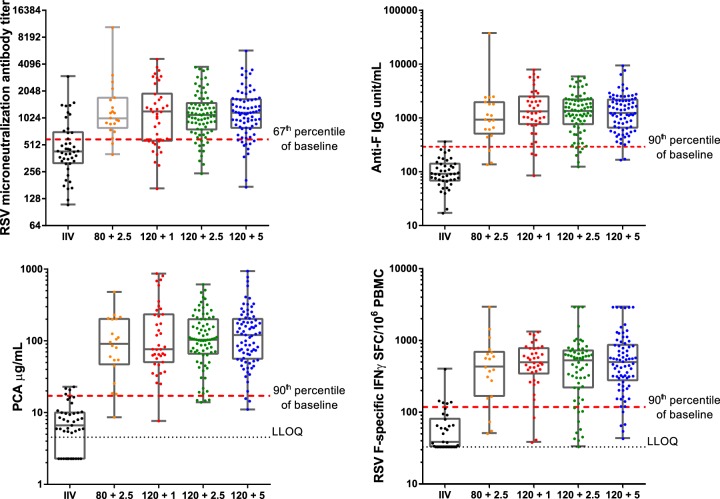
Immunogenicity of an RSV sF vaccine adjuvanted with GLA in 2% SE (GLA-SE) by dose. (A) RSV microneutralization antibody titer at postdose day 29; (B) anti-F IgG antibody levels (antibody units per milliliter) at postdose day 29; (C) palivizumab-competitive antibody levels (micrograms per milliliter) at postdose day 29; (D) F-specific gamma interferon ELISPOT assay results (number of spot-forming cells [SFC] per 10^6^ peripheral blood mononuclear cells [PBMC]) at postdose day 8. Bars, medians and first and third quartiles; black dotted lines, lower limit of quantitation (LLOQ); red dotted lines, 67th percentile of baseline microneutralizing antibody titers or 90th percentile of the baseline levels for the other assays. F IgG, F-specific IgG; PCA, palivizumab-competitive antibody; RSV, respiratory syncytial virus.

All subjects had detectable antibodies at the baseline, as measured in the RSV subtype A and B microneutralization (MN) and F IgG assays. In contrast, 45% and 40% of the subjects had baseline values below the lower limit of quantification (LLOQ) in enzyme-linked immunosorbent spot (ELISPOT) and palivizumab-competitive antibody (PCA) assays, respectively. Baseline values varied among the cohorts (RSV A MN assay geometric mean titer [GMT] range, 400 to 514). Influenza vaccine had no consistent effect on RSV vaccine immunogenicity (Fig. S2); therefore, data are presented for all subjects in a group regardless of the receipt of IIV. For all assays, the responses in the RSV vaccine groups were statistically significantly greater than those in control vaccine recipients (Fig. 1). Geometric mean fold rises (GMFRs) in RSV A MN assay responses on day 29 ranged from 2.41 to 2.84 across those receiving the various vaccine formulations, with the 95% confidence intervals (CIs) among the vaccine dose groups overlapping, but the 95% CIs for the controls (GMFR, 0.92) did not overlap those for the vaccine dose groups. The day 29 GMFRs for F IgG antibodies and PCAs were greater than those for MN assay antibodies; for example, GMFRs for subjects who received the 120 μg sF and 5 μg GLA formulation were 16.46, 23.71, and 2.63, for F IgG antibodies, PCAs, and MN assay antibodies, respectively. Cross-neutralizing antibodies to the RSV B 9320 strain were also generated (the day 29 GMFR in cohort 3 was 1.76 [95% CI, 1.51, 2.07], whereas it was 1.01 [95% CI, 0.94, 1.09] in IIV recipients). No subject who received a control vaccine had a ≥3-fold increase in antibody titers or T-cell responses.

**FIG 2:**
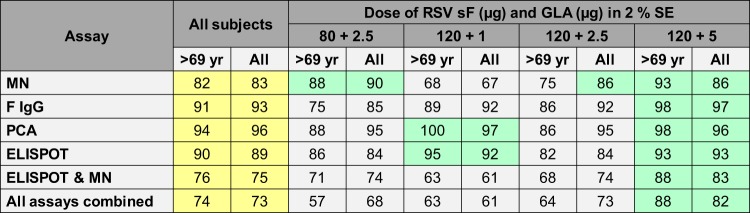
Percentage of subjects with day 29 humoral immunogenicity and day 8 F-specific gamma interferon ELISPOT assay results greater than the 67th percentile of the baseline titer (microneutralization assay) or 90th percentile of the baseline levels (all other assays). Values for all subjects combined have a yellow background; values less than the values for all subjects have a gray background, and values more than the values for all subjects have a green background. By cohort, results are presented for all subjects and for subjects older than the median age (69 years). ELISPOT, F-specific gamma interferon enzyme-linked immunosorbent spot assay; F IgG, F-specific IgG; GLA, glucopyranosyl lipid A; MN, microneutralization assay; PCA, palivizumab-competitive antibodies; RSV, respiratory syncytial virus; SE, stable emulsion; sF, soluble RSV fusion protein.

In the subset analysis, for all assays, those with the lowest baseline values had lower day 29 geometric mean values but greater GMFRs (Fig. S3 shows data for the RSV A MN and ELISPOT assays; similar results were observed in all assays [data not shown]). Age did not have a consistent effect on the responses (data not shown). The proportions of all subjects and of subjects >69 years of age who developed RSV A MN assay titers greater than the 67th percentile of the baseline titer and F IgG antibody and PCA responses and F-specific gamma interferon (IFN-γ) T-cell ELISPOT assay responses greater than the 90th percentile of the baseline responses are presented in Fig. 2. In this analysis, the greatest overall immunogenicity and the greatest immunogenicity in subjects >69 years of age were observed in subjects immunized with 120 μg of sF in 5 μg GLA in SE.

**FIG 3:**
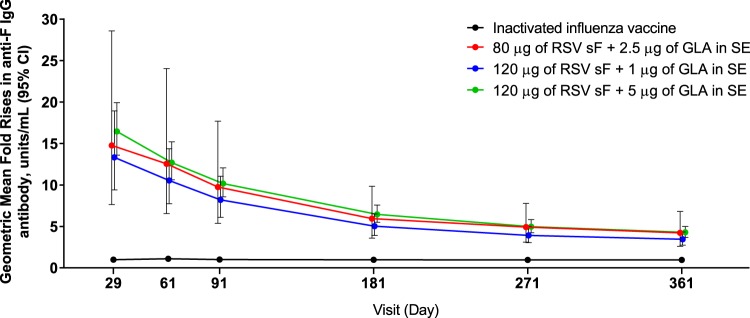
Anti-F immunoglobulin G antibody level over time in subjects who received either the RSV vaccine (by dose) or the influenza vaccine. The geometric mean fold rise in the antibody level from that at the baseline is presented by day postdose. CI, confidence interval; F IgG, F-specific IgG; GLA, glucopyranosyl lipid A; RSV, respiratory syncytial virus; SE, stable emulsion; sF, soluble RSV fusion protein.

Antibody levels declined over time, as measured in the F IgG assay (Fig. 3), but GMFRs were statistically significantly higher for all vaccine formulation groups than for the control group. For subjects who received the 120 μg sF and 5 μg GLA formulation, 84.2% (95% CI, 74.0, 91.6%) had antibody levels ≥3-fold greater than the baseline level on day 181, 73.6% (95% CI, 61.9, 83.3%) had such antibody levels on day 271, and 67.1% (95% CI, 55.1, 77.7%) had such antibody levels on day 361.

In an initial assessment of the potential for the RSV vaccine to interfere with the immunogenicity of IIV, the GMFR ratios of hemagglutination inhibition (HAI) antibodies for the H1N1, H3N2, and B strains were 1.13 (95% CI, 0.68, 1.87), 1.53 (95% CI, 0.86, 2.72), and 1.09 (95% CI, 0.76, 1.55), respectively, in cohort 2 subjects and 0.97 (95% CI, 0.54, 1.75), 1.28 (95% CI, 0.69, 2.38), and 1.00 (95% CI, 0.71, 1.40), respectively, in cohort 3 subjects. After adjustment for the baseline titer, all upper bounds of the 95% CI for the GMT ratio were <2, except for the H3N2 strain in cohort 2. The values for the H3N2 strain were 1.53 (95% CI, 1.06, 2.21) in cohort 2 and 0.85 (95% CI, 0.58, 1.24) in cohort 3.

## DISCUSSION

Data from this study confirm the immunogenicity and safety observed in the earlier study of the GLA-SE-adjuvanted RSV sF-based vaccine and also describe the safety and immunogenicity of vaccine candidates containing higher doses of both RSV sF and GLA (21). Although higher doses of GLA resulted in increased local reactogenicity, at the highest dose tested, reactogenicity events were low grade and similar in frequency to those occurring after immunization with IIV. Thus, selection of an RSV sF and GLA dose for evaluation in a phase 2 efficacy study was based on immunogenicity results.

Although the phase 2b study (ClinicalTrials.gov registration no. NCT02508194) failed to meet its primary efficacy endpoint, the detailed immunogenicity data from this phase 1b study are relevant to understanding the outcome of the phase 2b study and are relevant because other fusion protein-containing vaccines are being developed for the prevention of RSV in older adults. In addition, both the demonstration of local reactogenicity associated with the vaccine and the long-term safety follow-up provide support for the safety of the GLA-SE adjuvant, which is being used in other vaccines.

In all formulations tested, the RSV vaccine elicited immune responses that were statistically significantly greater than those elicited by control vaccines. For dose selection, results from 4 assays, the results of each of which were examined by several methods (geometric mean values, GMFR values, proportion of the population with levels above a prespecified cutoff whether by fold rise or proportion of the baseline, and others) led to a large number of comparisons. Because subjects were seropositive, imbalances in the baseline values across groups also contributed to the complexity, as did the fact that the differences between cohorts were small, probably because the doses studied resulted in nearly maximal responses. This led us to use the analysis for which the results are shown in Fig. 2, which focused on the subjects at the greatest risk on the basis of age or because they had the lowest baseline values and provided a visually interpretable summary of a large amount of data. The optimal cutoff value for analysis has yet to be defined, given the lack of an immune correlate of protection from RSV disease, but it is likely that a key success factor is the proportion of subjects whose postdose responses were above a specific value rather than the overall magnitude of the postdose responses, which would also represent increased immunogenicity in those who might have been protected at the baseline. This analysis lends itself to a population-based assessment of immunogenicity by age, which lessens the risk of selection of a dose that is inappropriate for the oldest and most immunosenescent people. In this study, older age did not have a readily discernible impact on immunogenicity across all assays, which could represent a benefit of the adjuvant (22).

In this study, the ability of a vaccine based on the F protein (in the postfusion configuration) from an RSV A strain to elicit antibodies to RSV B was demonstrated, confirming in humans what had previously been demonstrated in a nonhuman primate study (18). This generation of cross-neutralizing antibodies is expected, given the conserved nature of the F protein (20).

Data from this study are also useful to approach a booster dose strategy for this RSV vaccine. RSV is a seasonal illness, with a short, defined season in the temperate zone and a longer season in more southern parts of the United States, such as Florida (23). For a vaccine that is expected to be administered in autumn concurrently with the influenza vaccine and prior to the respiratory virus season, the most important long-term immunogenicity time points are those that occur through the end of the respiratory virus season. In our data, seroresponses remained substantial through 9 months postdose and declined through 1 year. Annual dosing is likely to be necessary, which would be consistent with the known short protection from reinfection that is afforded by infection with wild-type RSV A, but a requirement for annual reformulation due to a strain change is not expected (13, 14).

The primary limitations of this study are related to the small sample size of phase 1 studies. There was a clear effect of baseline values on postdose responses, and there were differences in baseline values among the cohorts. Sample sizes were too small to demonstrate statistically significant differences in immunogenicity between doses, although for all cohorts, the responses were statistically significantly greater than those for the control group. The sample size was also too small to conclusively establish a lack of an effect of the RSV vaccine on the immunogenicity of IIV. Nevertheless, the data established a reasonable likelihood that concurrent administration is safe in terms of reactogenicity and immunogenicity, which supported concomitant IIV use in the phase 2 study. A formal lack of interference in subjects at least 6 months from prior immunization with IIV will be evaluated in a larger study. In addition, very frail subjects, such as those in a nursing home, were not included, although the median age of the enrolled subjects was reasonably representative of that of the U.S. population over 60 years of age on the basis of census data. It is possible that age is not the most appropriate marker for the population at greatest risk of a poor immune response, although age has been used to establish a population that could benefit from the use of a high-antigen-dose influenza vaccine (24).

We used a single assay to examine cellular immunity, so our understanding of the RSV-specific T-cell response elicited is limited, but additional analysis of the T-cell responses is ongoing in a separate study. Our understanding of key humoral immune responses is also limited, although higher serum neutralizing antibody levels are known to correlate with a reduced risk for RSV infection, and the efficacy of passively administered anti-RSV antibodies in preventing disease in infants supports the importance of antibody responses (11, 12, 14, 25–29).

In conclusion, an experimental adjuvanted RSV F subunit vaccine designed to prevent RSV disease in an older adult population resulted in substantial humoral and cellular immune responses and acceptable safety when administered with placebo or IIV. Between the phase 1a study and this phase 1b study, substantial F antigen and adjuvant dose exploration was completed, permitting a single formulation to be tested in phase 2.

## MATERIALS AND METHODS

### Ethics statement.

The study (ClinicalTrials.gov registration no. NCT02289820) was carried out in accordance with the Declaration of Helsinki and good clinical practice guidelines. The study protocol and amendments and the subject informed consent document were approved by the Copernicus Group Independent Review Board and the Vanderbilt University Institutional Review Board. All subjects provided written informed consent.

### Study design.

**FIG 4:**
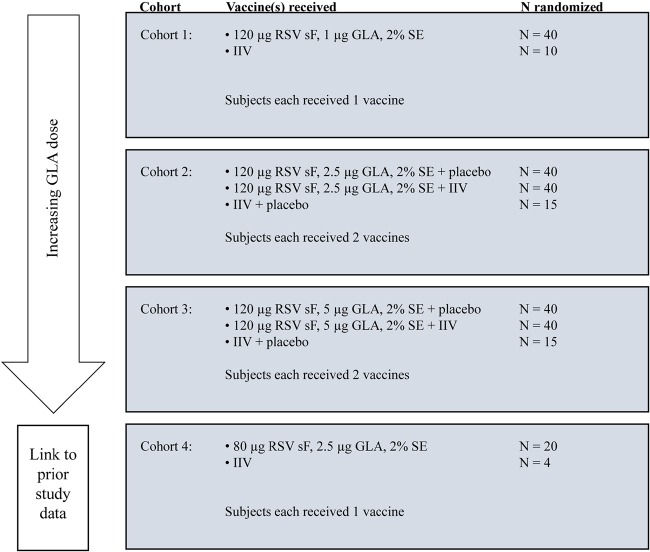
Study dosing cohorts. Subjects within each cohort were randomized to the investigational product arm and, for subjects receiving a vaccine in each arm, to the dosing arm. For analysis, all subjects receiving IIV (with or without placebo) were combined into a control group. GLA, glucopyranosyl lipid A; IIV, inactivated influenza vaccine; RSV, respiratory syncytial virus; SE, stable emulsion; sF, soluble RSV fusion protein.

In this phase 1b, double-blind, controlled, adjuvant-escalation (1.0, 2.5, and 5 μg of GLA) study conducted in adults ≥60 years of age, 264 subjects (healthy subjects or subjects with stable chronic illnesses) were to be enrolled at 5 centers in the United States in 2015. They were randomized by cohort, as described in Fig. 4, using an Internet-based interactive response system. Subjects received 1 vaccine in cohorts 1 and 4. For cohorts 2 and 3, in which the subjects received an RSV vaccine and either IIV or placebo to assess the safety and immunogenicity of concurrently administered IIV and RSV vaccines, the vaccines were randomly assigned to be administered in the right or the left arm. The dose of 80 μg of sF plus 2.5 μg of GLA was included to bridge to phase 1a study data. In a preplanned interim analysis, some investigators were made unblind to the treatments after the subjects had completed 90 days of safety follow-up, but site staff and subjects remained blind to the treatments until the end of the study (day 361).

### Subjects.

Subjects were required to be ≥60 years of age, to be not institutionalized or homebound, to weigh >40.9 kg, and to have met a gender-specific hemoglobin requirement. Major exclusions included an unstable medical condition or a recent change in therapy, receipt of influenza vaccine within the previous 60 days, receipt of products that could include exogenous antibodies, clinically significant abnormalities in screening tests, viral hepatitis, a history of or current autoimmune disorder other than hypothyroidism, immunosuppression, a body mass index of ≥40 kg/m^2^, or the taking of medications or the existence of conditions that could cause injection site bleeding.

### Study vaccines.

sF in the postfusion conformation (20) derived and modified from the sequence of an RSV A strain was expressed in a Chinese hamster ovary cell line. GLA-SE was provided by and licensed from Immune Design Corporation (Seattle, WA) pursuant to an existing agreement. RSV sF was from the same lot used in the phase 1a study (21); GLA-SE was remanufactured to use non-egg-derived phosphatidylcholine. Inactivated influenza vaccine (Fluzone) was purchased commercially and provided to the study sites. To prepare vaccine on-site, lyophilized RSV sF was reconstituted with sterile water and combined with liquid diluent, GLA-SE, and SE in a mixing vial to reach the concentrations of sF, GLA, and SE specified for each cohort. For all vaccines, a single 0.5-ml dose was drawn into a covered syringe to maintain the blind and administered into the deltoid muscle with a needle of the gauge and length described in previously published guidelines (30).

### Study assessments.

Specified solicited symptoms were recorded using a diary card between days 1 (dosing) and 7; AEs were recorded between day 1 and the day 29 visit; and serious AEs, new-onset chronic diseases, and AESIs, including events of potential autoimmune etiology, were recorded from days 1 to 361. Visits occurred on (approximately) days 1, 8, 29, 61, 91, 181, 271, and 361. Subjects were contacted by telephone at least monthly for safety follow-up. Toxicity grading was generally consistent with that on a standard table (31).

Vaccine immunogenicity was assessed as it was in the phase 1a study but with the addition of an assay to measure RSV B9320-neutralizing antibodies that was performed similarly to the assay used to measure RSV A-neutralizing antibodies, which were measured in heat-inactivated serum without added complement using green fluorescent protein-tagged RSV A2 as previously described (18). Anti-F IgG antibody levels were determined using a multiplexed assay based on the Meso Scale Discovery platform (Meso Scale Diagnostics, Gaithersburg, MD) that assessed antibodies to RSV sF and to nonvaccine antigens (RSV G protein from A and B strains and the RSV nucleoprotein [N]) as previously described (32). Palivizumab-competitive antibodies were assessed using an enzyme-linked immunosorbent assay (ELISA) in which biotin-labeled palivizumab mixed with serum samples was added to RSV F antigen-coated plates, and bound palivizumab was detected with horseradish peroxidase-conjugated streptavidin (21). F-specific IFN-γ enzyme-linked immunosorbent spot (ELISPOT) assay T-cell responses were measured using a peptide pool as previously described (33). All assays were performed at the baseline; the ELISPOT assay was also performed on day 8, and humoral assays were performed on day 29 in all subjects and on days 61, 91, 181, 271, and 361 in some subjects. The levels of RSV B-neutralizing antibodies in some subjects were assessed on days 1 and 29 using an assay similar to the RSV A MN assay. For results reported to be below the LLOQ, a value equal to half of the LLOQ was imputed for calculation. The LLOQ for the microneutralization assays (log_2_ scale), anti-F IgG antibody and palivizumab-competitive antibody levels, and HAI results were 3.32, 0.66 antibody unit/ml, 4.55 μg/ml, and 10, respectively. For the ELISPOT assay, if the count was reported to be too many to count, the imputed value was the upper bound of individual replicates + 1 (i.e., 2,963.7 + 1 = 2,964.7 spot-forming cells/10^6^ peripheral blood mononuclear cells). After subtraction of the read for the mock-infected serum from the read for RSV F, for counts of less than 33.3, a value of 33.3 (the LLOQ) was assigned for analysis. Seroresponse data are presented as a ≥3-fold rise from the baseline. On the basis of the precision of the assays, there was a <5% possibility that a 3-fold rise was due to chance. The levels of anti-Ga and anti-Gb antibodies (the RSV A and B subtypes of the RSV G protein, respectively) and anti-N antibodies were assessed at each immunogenicity time point using a multiplexed assay (32). Subjects with a ≥4-fold rise in the levels of IgG antibodies to Ga, Gb, or N were considered to have been exposed to wild-type RSV, and their vaccine immunogenicity data from that time point onward were excluded.

The potential effect of the adjuvanted RSV vaccine on antibody responses to an IIV (Fluzone) was assessed in samples obtained on days 1 and 29 using an HAI assay (Focus Diagnostics, Inc., Cypress, CA).

### Statistical analysis.

The sample size was selected to provide an initial assessment of safety and immunogenicity. Missing data were not imputed, and formal statistical comparisons among groups were not performed. Nonoverlapping 95% CIs determined statistical significance. For evaluating potential vaccine interference, the sample size provided an 83% power to demonstrate that the per strain HAI GMTs in the concomitant RSV vaccine group were not inferior to those in the IIV control group on the basis of a 2-sample *t* test assuming a log normal distribution of HAI titers, a 1-sided alpha value of 0.1, a standard deviation for the HAI GMT of 1.4 in natural log transformation, no true HAI GMT difference between 2 groups, a noninferiority margin of 2-fold, and 0% attrition by day 29. Because of variability in the baseline HAI values, an *ad hoc* sensitivity analysis was conducted to reestimate the GMT ratio by adjusting for the baseline titer using an analysis of covariance model with treatment as the fixed effect and the baseline titer as a covariate.

A prespecified comparison of the proportion of subjects with postdose values above the 67th percentile of the baseline, the value used in a previous study, was performed (34). In an *ad hoc* analysis, the 90th percentile of the baseline distribution, which approximated the maximum separation of pre- and postdose distributions, was used for the comparison.

## Supplementary Material

Supplemental material
