# Supplementary material for: Dose Selection for an Adjuvanted Respiratory Syncytial Virus F Protein Vaccine for Older Adults Based on Humoral and Cellular Immune Responses
Source: Clin Vaccine Immunol. 2017 Sep 5;24(9):e00157-17. doi: 10.1128/CVI.00157-17 (PMC5585697; doi:10.1128/CVI.00157-17)
Supplement: Supplemental material [file supp_24_9_e00157-17__index.html]

Supplemental material 

# Dose Selection for an Adjuvanted Respiratory Syncytial Virus F Protein Vaccine for Older Adults Based on Humoral and Cellular Immune Responses

## Supplemental material

- Supplemental file 1 -

  Fig. S1. CONSORT diagram for phase 1b study of an adjuvanted RSV vaccine. Fig. S2. Immune responses in subjects in cohorts 2 and 3 who received RSV vaccine with placebo or with IIV. Fig. S3. Effect of baseline on (A) day 29 microneutralizing antibody titers, (B) day 8 F-specific IFN-γ T cell ELISPOT counts, (C) day 29 fold rise in microneutralizing antibodies, and (D) day 8 fold rise in F-specific IFN-γ T cell ELISPOT counts. Table S1. Demographics of subjects dosed. Table S2. Systemic solicited symptoms reported during days 1 to 7. Table S3. Treatment-emergent new-onset chronic diseases and serious adverse events during days 1 to 361.

  PDF, 650K
