# Supplementary material for: Dose Selection for an Adjuvanted Respiratory Syncytial Virus F Protein Vaccine for Older Adults Based on Humoral and Cellular Immune Responses
Source: Clin Vaccine Immunol. 2017 Sep 5;24(9):e00157-17. doi: 10.1128/CVI.00157-17 (PMC5585697; doi:10.1128/CVI.00157-17)
Supplement: Supplemental material [file CVI.00157-17_zcd999095513s1.pdf]

Supplementary figure legends

Supplementary Figure 1. CONSORT diagram for phase 1b study of an adjuvanted RSV vaccine. GLA = glucopyranosyl lipid A; IIV = inactivated influenza vaccine; LTFU = lost to follow-up; sF = soluble F protein; WD = withdrew.

Supplementary Figure 2. Immune responses in subjects in cohorts 2 and 3 who received RSV vaccine with placebo (solid bars) or with IIV (hatched bars). A) Microneutralizing antibody on day 29; top figure, geometric mean titers; bottom figure, geometric mean fold rises from baseline. B) F-specific interferon-gamma ELISPOT assay on day 8; top figure, geometric mean counts; bottom figure, geometric mean fold rises from baseline. CI = confidence interval; GLA-SE = glucopyranosyl lipid A in 2% stable emulsion; IIV = inactivated influenza; PBMC = peripheral blood mononuclear cells; RSV = respiratory syncytial virus; sF = soluble F protein; SFC = spot-forming cells.

Supplementary Figure 3. Effect of baseline on A) day 29 microneutralizing antibody titers, B) day 8 F-specific IFN-γ T cell ELISPOT counts; C) day 29 fold rise in microneutralizing antibodies, and D) day 8 fold rise in F-specific IFN-γ T cell ELISPOT counts. CI = confidence interval; GLA-SE = glucopyranosyl lipid A in 2% stable emulsion; IIV = inactivated influenza; RSV = respiratory syncytial virus; sF = soluble F protein.

Supplementary Figure 1.

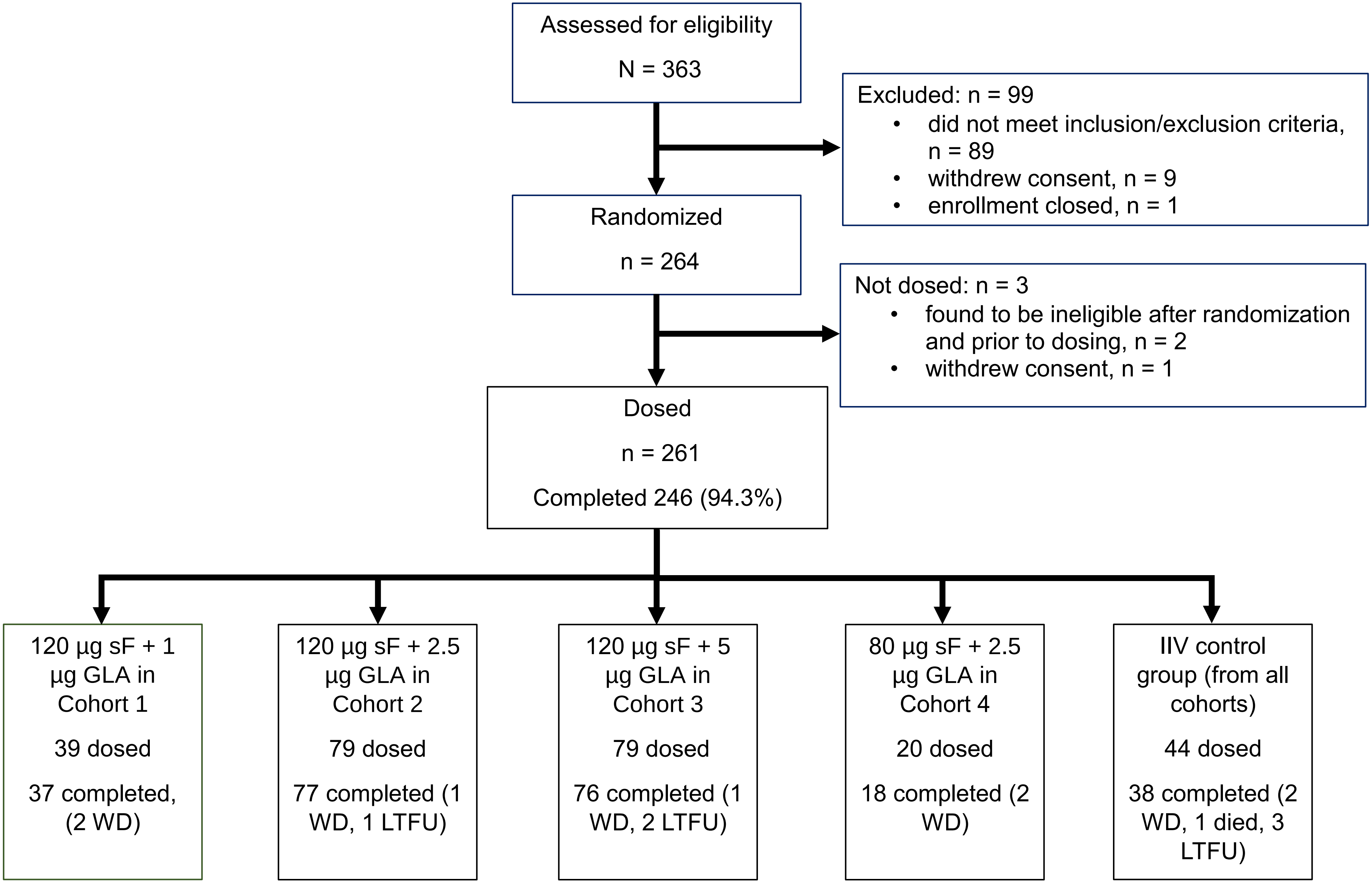

Supplementary Figure 2.

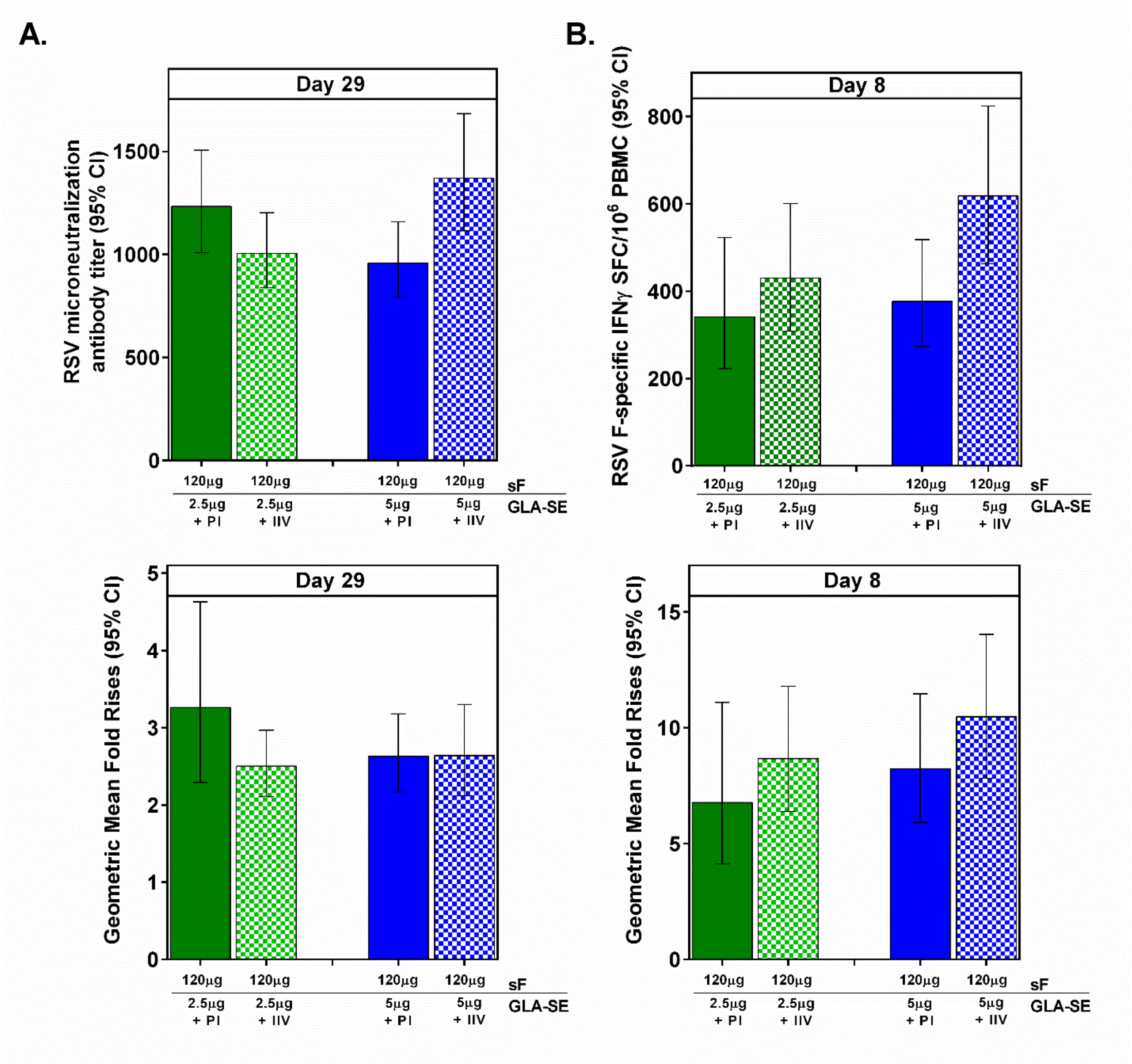

Supplementary Figure 3.

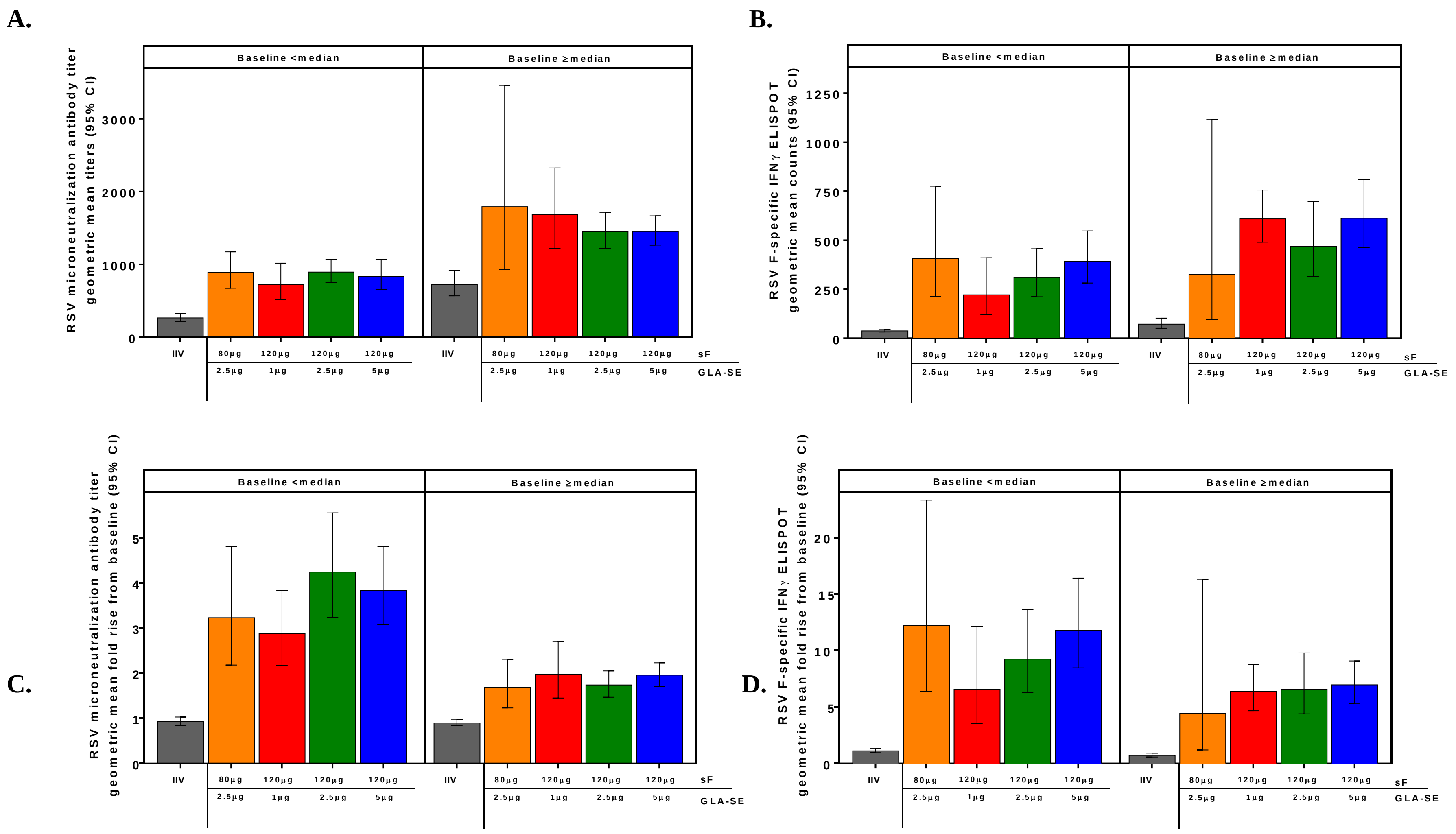

Supplementary Table 1. Demographics of subjects dosed.

Characteristic	IIV (Alone or with placebo) N = 44	Cohort 4 80 µg sF + 2.5 µg GLA N = 20	Cohort 1 120 µg sF + 1 µg GLA N = 39	Cohort 2 120 µg sF + 2.5 µg GLA (with either IIV or placebo) N = 79	Cohort 3 120 µg sF + 5 µg GLA (with either IIV or placebo) N = 79	Total N = 261
Median age (range), y	67 (60, 91)	67 (60, 80)	69 (60, 86)	68 (60, 86)	70 (60, 89)	68 (60, 91)
Age >69, y (%)	34.1	40	48.7	36.7	50.6	42.5
Male, (%)	43.2	35.0	46.2	41.8	53.2	45.6
Hispanic or Latino ethnicity, (%)	15.9	25.0	33.3	12.7	16.5	18.4
Race						
Black or African American	6.8	5.0	5.1	5.1	3.8	5.0
Other	2.3	5.0	0	3.8	1.3	1.5
White	90.9	90.0	94.9	91.1	94.9	92.7

GLA = glucopyranosyl lipid A; IIV = inactivated influenza vaccine; sF = soluble respiratory syncytial fusion protein.

Other includes subjects not in listed categories, including if multiple categories checked.

Supplementary Table 2. Systemic solicited symptoms reported during days 1 to 7.

Solicited Symptom	IIV (Alone or with placebo) N = 44	Cohort 4 80 µg sF + 2.5 µg GLA N = 20	Cohort 1 120 µg sF + 1 µg GLA N = 39	Cohort 2 120 µg sF + 2.5 µg GLA (with either IIV or placebo) N = 78	Cohort 3 120 µg sF + 5 µg GLA (with either IIV or placebo) N = 78
	n (%)	n (%)	n (%)	n (%)	n (%)
Any solicited symptom[a]	28 (63.6)	12 (60.0)	14 (35.9)	52 (66.7)	56 (71.8)
Any systemic solicited symptom	11 (25.0)	6 (30.0)	10 (25.6)	23 (29.5)	22 (28.2)
Fatigue/tiredness	8 (18.2)	3 (15.0)	4 (10.3)	18 (23.1)	18 (23.1)
Generalized muscle aches	4 (9.1)	0	7 (17.9)	8 (10.3)	10 (12.8)
Headache	9 (20.5)	4 (20.0)	6 (15.4)	9 (11.5)	6 (7.7)
Fever ≥38 °C	0	0	0	0	2 (2.6)

GLA = glucopyranosyl lipid A; IIV = inactivated influenza vaccine; sF = soluble respiratory syncytial fusion protein.

Subjects were counted once for each solicited symptom regardless of the number of events.

[a]Any symptom includes systemic and local symptoms.

Supplementary Table 3. Treatment-emergent new onset chronic diseases and serious adverse events during days 1 to 361.

Type of event	Vaccine (s) received	Event[a]	Subject age (years), sex	Event grade	Day of onset postdose	Relationship to study dosing
**New onset chronic disease**	**80 µg RSV sF + 2.5 µg GLA**	Haemoglobin decreased	66, Male	1	216	Not related
	**120 µg RSV sF + 1 µg GLA**	Benign prostatic hyperplasia	65, Male	1	243	Not related
	**120 µg RSV sF + 2.5 µg GLA and IIV**	Anxiety	67, Female	1	207	Not related
		Anxiety	60, Male	2	~4 months	Not related
**Serious adverse event**	**IIV + Placebo**	Cardiac arrest	79, Male	5 (death)	320	Not related
	**120 µg RSV sF + 1 µg GLA**	Rectal adenocarcinoma	84, Female	3	255	Not related
	**120 µg RSV sF + 2.5 µg GLA and placebo**	Breast cancer female	62, Female	3	23	Not related
		Arthralgia	75, Female	2	75	Not related

Type of event	Vaccine (s) received	Event[a]	Subject age (years), sex	Event grade	Day of onset postdose	Relationship to study dosing
	**120 µg RSV sF + 5 µg GLA and IIV**	Invasive ductal breast carcinoma	69, Female	3	58	Not related

GLA = glucopyranosyl lipid A; IIV = inactivated influenza vaccine; sF = soluble respiratory syncytial fusion protein.

RSV sF + GLA vaccines also contained 2% stable emulsion.

[a]Coded term, Medical Dictionary for Regulatory Activities Version 17.0, preferred term.
